# N-Linked Glycosylation and Expression of Duck Plague Virus pUL10 Promoted by pUL49.5

**DOI:** 10.1128/spectrum.01625-23

**Published:** 2023-06-28

**Authors:** Chunmei Li, Mingshu Wang, Anchun Cheng, Ying Wu, Bin Tian, Qiao Yang, Qun Gao, Di Sun, Shaqiu Zhang, Xumin Ou, Yu He, Juan Huang, Xinxin Zhao, Shun Chen, Dekang Zhu, Mafeng Liu, Renyong Jia

**Affiliations:** a Institute of Veterinary Medicine and Immunology, College of Veterinary Medicine, Sichuan Agricultural University, Chengdu City, Sichuan, China; b Key Laboratory of Animal Disease and Human Health of Sichuan Province, Sichuan Agricultural University, Chengdu City, Sichuan, China; c Avian Disease Research Center, College of Veterinary Medicine, Sichuan Agricultural University, Chengdu City, Sichuan, China; d Engineering Research Center of Southwest Animal Disease Prevention and Control Technology, Ministry of Education of the People's Republic of China, Chengdu City, Sichuan, China; University of Florida College of Dentistry

**Keywords:** alphaherpesviruses, duck plague virus, pUL10, N-linked glycosylation, pUL49.5

## Abstract

Duck plague virus (DPV) is a member of the alphaherpesvirus subfamily, and its genome encodes a conserved envelope protein, protein UL10 (pUL10). pUL10 plays complex roles in viral fusion, assembly, cell-to-cell spread, and immune evasion, which are closely related to its protein characteristics and partners. Few studies have been conducted on DPV pUL10. In this study, we identified the characteristics of pUL10, such as the type of glycosylation modification and subcellular localization. The characteristic differences in pUL10 in transfection and infection suggest that there are other viral proteins that participate in pUL10 modification and localization. Therefore, pUL49.5, the interaction partner of pUL10, was explored. We found that pUL10 interacts with pUL49.5 during transfection and infection. Their interaction entailed multiple interaction sites, including noncovalent forces in the pUL49.5 N-terminal domains and C-terminal domains and a covalent disulfide bond between two conserved cysteines. pUL49.5 promoted pUL10 expression and mature N-linked glycosylation modification. Moreover, deletion of UL49.5 in DPV caused the molecular mass of pUL10 to decrease by approximately3 to 10 kDa, which suggested that pUL49.5 was the main factor affecting the N-linked glycosylation of DPV pUL10 during infection. This study provides a basis for future exploration of the effect of pUL10 glycosylation on virus proliferation.

**IMPORTANCE** Duck plague is a disease with high morbidity and mortality rates, and it causes great losses for the duck breeding industry. Duck plague virus (DPV) is the causative agent of duck plague, and DPV UL10 protein (pUL10) is a homolog of glycoprotein M (gM), which is conserved in herpesviruses. pUL10 plays complex roles in viral fusion, assembly, cell-to-cell spread, and immune evasion, which are closely related to its protein characteristics and partners. In this study, we systematically explored whether pUL49.5 (a partner of pUL10) plays roles in the localization, modification, and expression of pUL10.

## INTRODUCTION

The *Herpesviridae* family contains multiple viruses that cause a variety of infections in diverse species, and the alphaherpesvirus subfamily can establish lifelong latent infection in neurons (1, 2). Alphaherpesviruses consist of a lipid envelope, a protein-rich tegument, a fixed morphological capsid, and a vital double-stranded DNA genome (3). Duck plague virus (DPV), also called anatid herpesvirus 1, is a member of alphaherpesviruses reported by the ICTV (4). The DPV genome has 78 open reading frames, and the proteins encoded by them are employed to take part in viral replication and morphology (4).

The envelope in alphaherpesviruses contains approximately 10 glycosylated proteins, which are vital for attachment, penetration, envelopment, and viral fusion (5–9). DPV protein UL10 (pUL10, glycoprotein M homolog) is encoded by the UL10 gene. It is conserved throughout the *Herpesviridae* family and nonessential for most alphaherpesviruses, such as herpes simplex virus 1 (HSV-1), bovine herpesvirus 1 (BHV-1), pseudorabies virus (PRV), and varicella-zoster virus (VZV) (10–13). pUL10 plays complex roles in viral fusion, assembly, cell-to-cell spread, and immune evasion, which are closely related to its protein characteristics and numerous partners (11, 13–21). pUL10 homologs have been shown to effectively help equine herpesviruses 1 (EHV-1) virions invade primary equine respiratory epithelial cells (22). HSV-1 pUL10 has been targeted to the inner nuclear membrane at 4 h after virus infection, and it may modulate the entry of endonuclear capsids into the cytoplasm via primary envelopment and development (20). Moreover, pUL10 has been found to be localized to and to attract glycoprotein D (gD), gH, and gL transport to the Golgi apparatus in PRV and HSV-1 (23). gH/gL was shown to be relocalized to the sites of secondary envelopment that were vital for their incorporation into virions (24). Furthermore, the location transfer of the fusion core protein by pUL10 may result in the inhibition of viral fusion (23). Additionally, pUL10 homologs of PRV, EHV-1, and infectious laryngotracheitis virus have been shown to have inhibitory effects on viral fusion in transfection (25). pUL10 is a type III transmembrane protein with eight transmembrane domains that are sufficient for its localization and viral growth (26). Two amino acids (aa; valine 42 and glycine 301) of pUL10 were found to be responsible for pUL10 maturation in VZV infection (10). Moreover, deletion of the UL10 gene of BHV-1 has been shown to reduce the plaque morphology (12), and the pUL10 and pUL11 double deletion mutant has been found to exhibit additive growth defects and 70% plaque size reduction in HSV-1 (13). When a pUL10 deletion mutant of EHV-1 was used as a live vaccine to immunize foals, the host produced significantly more complement and neutralizing antibodies than other vaccination groups (27).

pUL10 has multiple partners at different stages in viral infection. Glycoprotein N partners with pUL10 to modulate its activity in membrane fusion (17) and has been shown to act as an inhibitor of the transporter associated with antigen processing (TAP) to escape the recognition of cytotoxic T lymphocytes in BHV-1, PRV, and EHV-1 (28, 29). pUL10 interacts with gE and pUL49, which play a role in secondary envelopment and pUL49.5 incorporation (30). Extended-synaptotagmin 1, as an interaction partner of pUL10, is a key mediator that triggers the secretion of cytosolic protein (31), and its knockdown in host cells results in increased virus-induced fusion and infectious virions in supernatants (19). The XPO6 exportin is required for pUL10 to leave the nuclear membrane in late infection (16). In Epstein-Barr virus, pUL10 interacts with the cellular protein p32, which is involved in nuclear egress and promotes viral morphology (32). Although it is known that pUL10 has many partners, the effect of interacting proteins on pUL10 itself has not been specifically explored. In this study, we found that the characteristic difference of pUL10 in transfection and infection was prompted by pUL49.5. Moreover, pUL10 modification and expression facilitated by pUL49.5, pUL10 (cysteine 54 [C54]), and pUL49.5 (cysteine 46 [C46]) were vital for the interaction between them, with other interaction sites strengthening their connection. This study provides a basis for future exploration of the effect of pUL10 on virus proliferation.

## RESULTS

### Characteristics of DPV pUL10 localization and modification.

**FIG 1:**
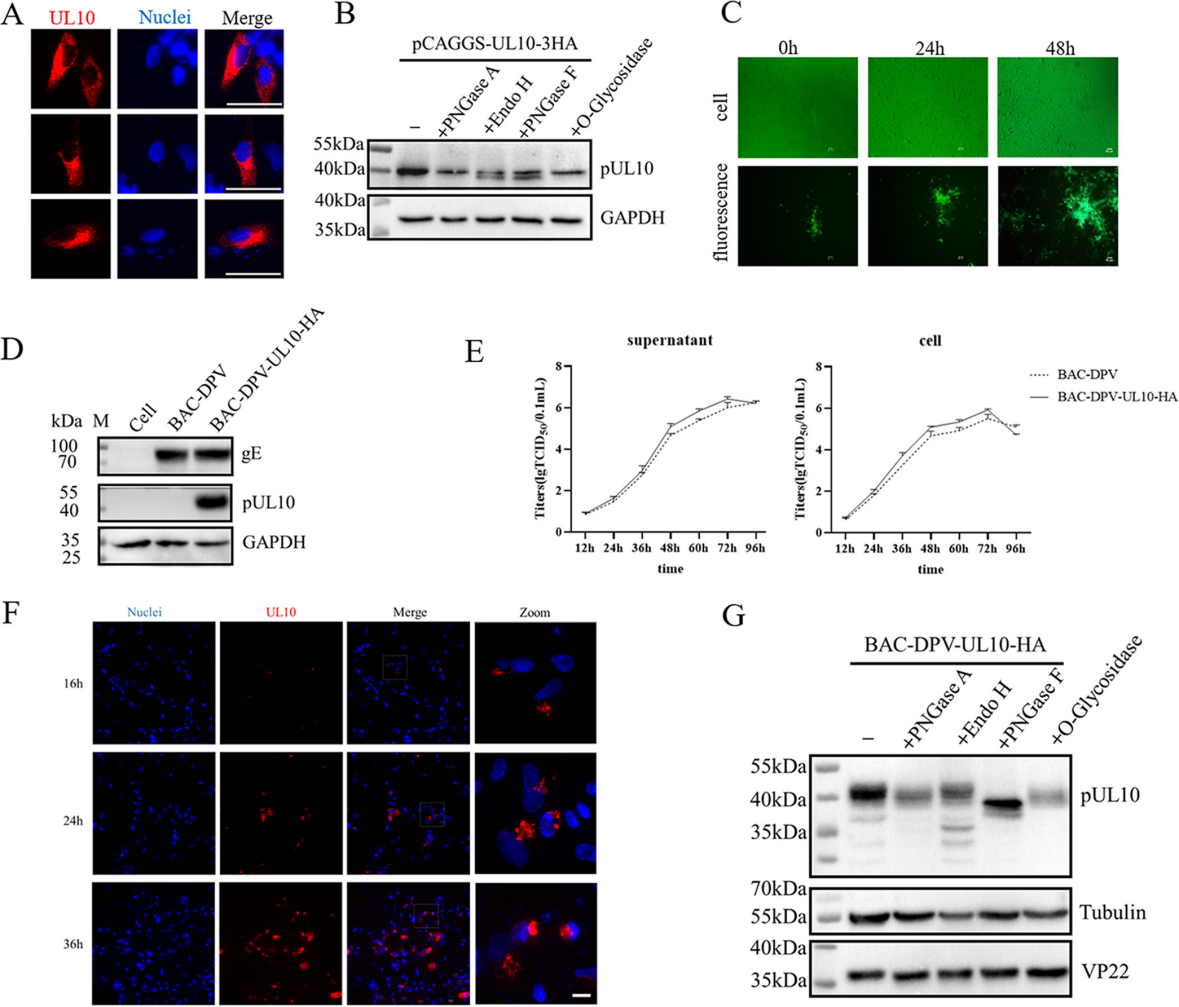
Localization and posttranscriptional modification of pUL10 in DEFs. (A) DPV pUL10 was localized in the cytoplasm. DEFs were transfected with 1,600 ng of pCAGGS-UL10-3HA, and cells were harvested at 36 h posttransfection. pUL10 was labeled with rabbit anti-HA tag polyclonal antibody and Alexa Fluor 568-conjugated goat anti-rabbit IgG (magnification, ×600; scale bar, 10 μm). (B) Type of pUL10 glycosylation when expressed alone. DEFs were transfected with pCAGGS-UL10-3HA. Cell lysates were collected 48 h posttransfection and divided into five equal parts to be treated with or without glycosidase. All samples were analyzed by WB. GAPDH was used as a loading control. M, protein marker. (C) Rescue of BAC-DPV-UL10-HA. Recombinant plasmids were transfected into DEFs, and green fluorescent plaques and corresponding cells were observed at 4, 5, and 6 days posttransfection (magnification, ×100; scale bar, 50 μm). (D) Identification of BAC-DPV-UL10-HA by WB analysis. DEFs were infected with BAC-DPV-UL10-HA and BAC-DPV (MOI of 1). Cell lysates were collected at 48 h postinfection. Mouse anti-HA tag MAb was used to detect pUL10. gE was used as a viral infection control. GAPDH was used as a loading control. M, protein marker. (E) Growth kinetics of BAC-DPV-UL10-HA. DEFs were infected with 100 TCID_50_ BAC-DPV-UL10-HA and BAC-DPV. Viral titers of cells and supernatants were detected at 12, 24, 36, 48, 60, 72, and 96 h postinfection. (F) Localization of DPV pUL10 in infection. DEFs were infected with BAC-DPV-UL10-HA (MOI of 0.1). DEFs were collected at 16, 24, and 36 h postinfection (magnification, ×400; scale bar, 10 μm). (G) Type of pUL10 glycosylation in infection. DEFs were infected with BAC-DPV-UL10-HA (MOI of 1). Cell lysates were collected at 48 h postinfection and divided into five equal parts to be treated with or without glycosidase. All samples were analyzed by WB. β-Tubulin was used as a loading control. VP22 was used as a viral infection control. M, protein marker.

First, pUL10 localization and posttranscriptional modification were explored. Duck embryo fibroblast cells (DEFs) in 12-well plates were transfected with pCAGGS-UL10-3HA and collected at 36 h posttransfection, and an indirect immunofluorescence assay (IFA) was performed. pUL10 was localized in the cytoplasm, but it was not uniformly distributed, and the red fluorescence was intense around the nucleus (Fig. 1A). To determine the type of pUL10 glycosylation, peptide-N-glycosidase A (PNGase A), peptide-N-glycosidase F (PNGase F), endoglycosidase H (Endo H), and O-glycosidase were used. The first three enzymes were targeted for N-linked glycosylation, and the last enzyme was targeted for O-linked glycosylation. Proteins with mature N-linked glycosylation were sensitive to PNGase A and PNGase F, which can cleave between the innermost *N*-acetylglucosamine and asparagine residues of high-mannose, hybrid, and complex oligosaccharides. PNGase A differs from PNGase F in that it cleaves N-linked glycans with or without α-(1, 3)-linked core fucose residues. Immature N-linked glycosylation was formed in the endoplasmic reticulum (ER), which was mainly cleaved by Endo H. When pUL10 was expressed alone in DEFs, it was partially digested by PNGase F and Endo H (Fig. 1B), which was consistent with its localization (Fig. 1A).

Next, the localization of pUL10 in DPV-infected cells was explored. Since there were no antibodies available for the IFA, a BAC-DPV-UL10-HA recombinant virus (where BAC is the bacterial artificial chromosome and HA is small peptide tag; see Materials and Methods for details) was constructed. DEFs were transfected with BAC-DPV-UL10-HA recombinant plasmids, and the changes in fluorescent plaques and cells over 2 days are shown in Fig. 1C. The HA tag sequence was successfully inserted into the carboxyl terminus of the UL10 gene, as expected by sequencing, which implied that the BAC-DPV-UL10-HA virus was generated. The pUL10-HA fusion protein was detected by mouse anti-HA tag MAb (M-HA), which showed that the HA tag was expressed correctly (Fig. 1D). The growth kinetics of BAC-DPV-UL10-HA were measured by a 50% tissue culture infective dose (TCID_50_) assay, which is described in Materials and Methods. As shown in Fig. 1E, according to the multistep growth curve, the HA tag did not affect viral replication in DEFs. These experimental results suggested that BAC-DPV-UL10-HA could be used in subsequent experiments. DEFs were infected with BAC-DPV-UL10-HA (multiplicity of infection [MOI] of 0.1) and collected at 16, 24, and 36 h postinfection, and IFA was performed. pUL10 was localized in the perinuclear region with punctate aggregation (Fig. 1F), which was different from the results shown in Fig. 1A. Moreover, DEFs were infected with BAC-DPV-UL10-HA (MOI of 1), and cell debris was collected at 48 h postinfection and prepared for the glycosidase treatment assay. PNGase F worked more efficiently than the other three enzymes to digest pUL10 in viral infection (Fig. 1G), and VP22 was a nonglycosylated protein and used as a viral infection control.

### DPV pUL49.5 interacts with pUL10 and promotes its glycosylation.

**FIG 2:**
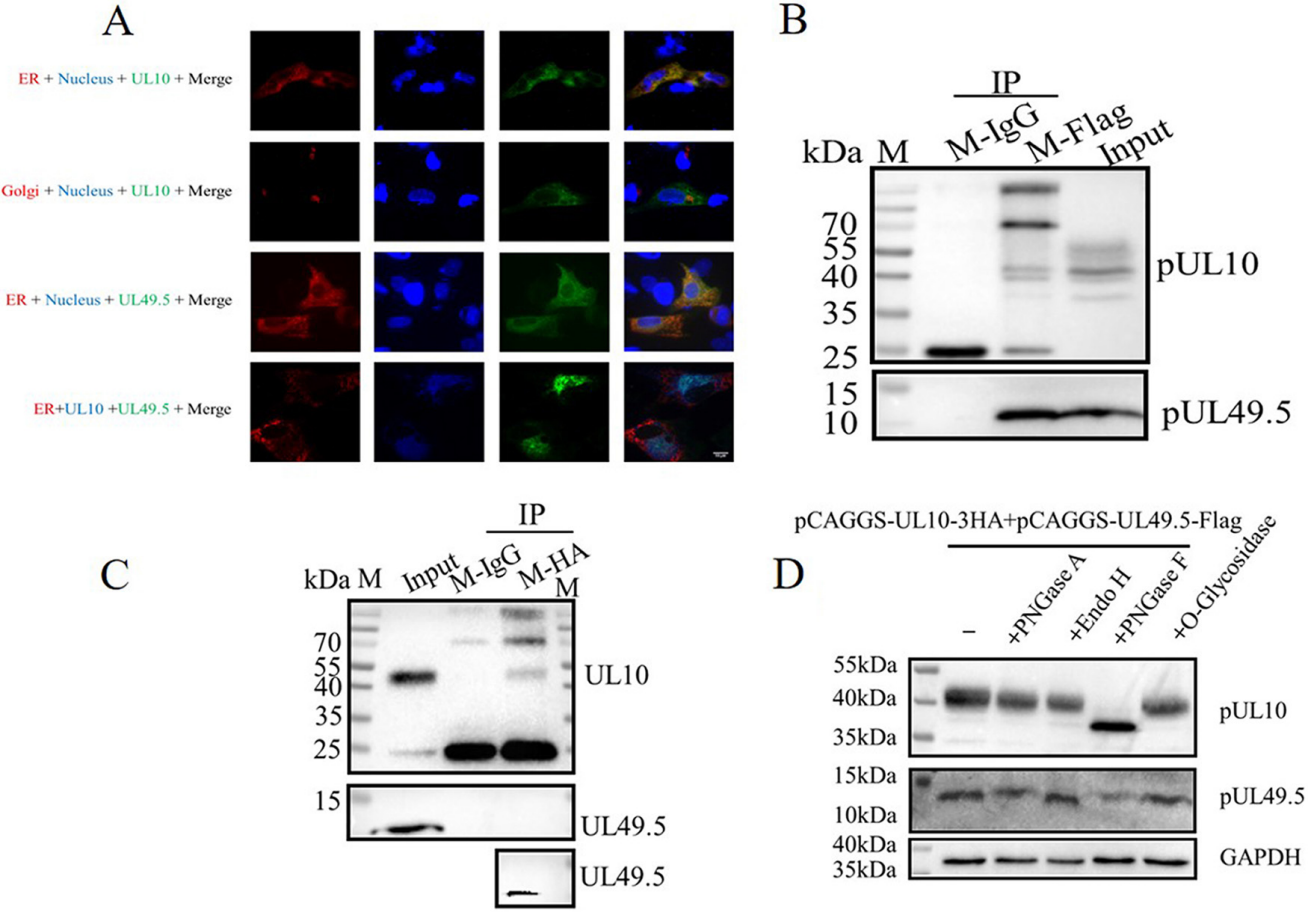
pUL10 interacted with pUL49.5 during transfection and infection. (A) Subcellular localization of pUL49.5 and pUL10. pDsRed2-ER plasmids were transfected into DEFs to mark the ER. The Golgi apparatus was labeled with mouse anti-GM130 and red fluorescence. DAPI was used to stain the nuclei. The use of antibodies is described in Materials and Methods. The pictures are arranged as indicated by the text on the left, and the colors of the text correspond to the images (magnification, ×400; scale bar, 10 μm). (B) Interaction of pUL49.5 and pUL10 in transfection. HEK-293T cells were cotransfected with pCAGGS-UL49.5-Flag and pCAGGS-UL10-3HA plasmids, M-Flag was added to the cell debris to precipitate pUL49.5, and M-IgG was used as a control. The WB shows that pUL10 was detected by M-HA and HRP-conjugated goat anti-mouse IgG light chain secondary (LCS) antibody and pUL49.5 was detected by M-Flag and HRP-conjugated goat anti-mouse IgG. (C) Interaction of pUL49.5 and pUL10 in viral infection. DEFs were infected with BAC-DPV-UL10-HA (MOI of 1). M-HA was added to cell debris to precipitate pUL10, and M-IgG was used as a control. The WB shows that pUL10 was measured by M-HA and HRP-conjugated goat anti-mouse IgG LCS. pUL49.5 was measured by R-UL49.5 and HRP-conjugated goat anti-rabbit IgG. (D) Type of pUL10 glycosylation when it is coexpressed with pUL49.5. DEFs were cotransfected with pCAGGS-UL49.5-Flag and pCAGGS-UL10-3HA. Cell lysates were collected and divided into five equal parts to be treated with or without glycosidase. All samples were analyzed by WB. GAPDH was used as a loading control. M-HA, mouse anti-HA tag MAb; M-Flag, mouse anti-Flag tag MAb; M-IgG, mouse IgG; R-UL49.5, rabbit anti-pUL49.5 polyclonal antibody. M, protein marker.

The difference in pUL10 characteristics in transfection and infection suggested that other viral proteins participated in assisting in the modification and localization of pUL10 (Fig. 1). Our laboratory previously reported that DPV pUL49.5 colocalized with pUL10 (33). Therefore, pUL49.5, as the first candidate partner of pUL10, and its subcellular localization were explored. DEFs were transfected with pDsRed2-ER and pCAGGS-UL10-3HA, and pUL10 was mainly localized in the ER. Moreover, pUL49.5 was localized in the ER when it was expressed alone, but pUL49.5 and pUL10 were able to leave the ER and accumulate in the perinuclear region when they were coexpressed (Fig. 2A). Therefore, we inferred that there would be an interaction between DPV pUL49.5 and pUL10. pCAGGS-UL49.5-Flag and pCAGGS-UL10-3HA plasmids were cotransfected into HEK-293T cells to explore their interaction. pUL49.5 was immunoprecipitated by mouse anti-Flag tag MAb (M-Flag), and pUL10 was also coimmunoprecipitated (Fig. 2B) but not in the mouse IgG (M-IgG) group. Additionally, mouse anti-HA tag mAb (M-HA) was immunoprecipitated with pUL10 and coimmunoprecipitated with pUL49.5 but not the M-IgG control group (Fig. 2C) in BAC-DPV-UL10-HA-infected (MOI of 1) DEFs. Then, when pUL10 was coexpressed with pUL49.5 in DEFs, it was mainly digested by PNGase F (Fig. 2D), which was similar to the results shown in Fig. 1G. Taken together, these data showed that the interaction between pUL49.5 and pUL10 was present in transfection and viral infection. Relocalization and mature N-linked glycoprotein of pUL10 were facilitated by pUL49.5.

### DPV pUL49.5 and pUL10 form a complex through multiple sites.

**FIG 3:**
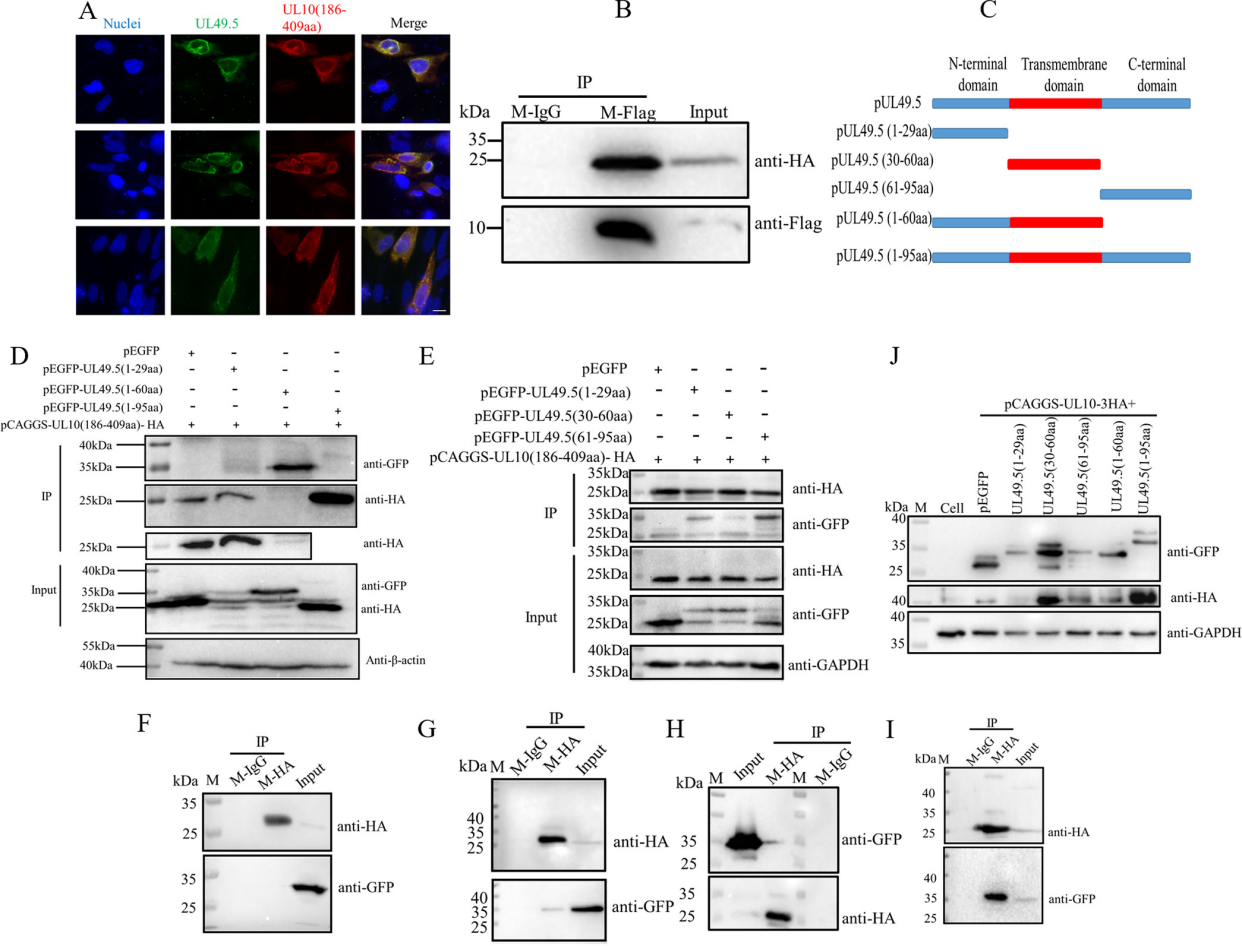
Interaction of pUL49.5 and pUL10(186–409 aa). (A) Colocalization of pUL49.5 and pUL10(186–409 aa). pUL10(186–409 aa) was labeled with R-HA and Alexa Fluor 568-conjugated goat anti-rabbit IgG. pUL49.5 was labeled with M-Flag and Alexa Fluor 488-conjugated goat anti-mouse IgG. DAPI was used to stain nuclei (magnification, ×400; scale bar, 10 μm). (B) Interaction of pUL49.5 and pUL10(186–409 aa). HEK-293T cells were cotransfected with pCAGGS-UL49.5-Flag and pCAGGS-UL10(186–409 aa)-HA plasmids. M-Flag was added to the cell debris to precipitate pUL49.5, and M-IgG was used as a control. The WB shows that pUL49.5 was detected by M-Flag and HRP-conjugated goat anti-mouse IgG secondary antibody, and pUL10(186–409 aa) was labeled by R-HA and HRP-conjugated goat anti-rabbit IgG heavy chain secondary (HCS) antibody. (C) Schematic diagram of truncated pUL49.5. (D and E) Interaction of truncated pUL49.5 and pUL10(186–409 aa). HEK-293T cells were cotransfected with relevant plasmids (shown in picture). M-HA was added to cell debris to precipitate pUL10(186–409 aa). The WB shows that truncated pUL49.5 or pEGFP was detected by rabbit anti-GFP monoclonal antibody (R-GFP) and HRP-conjugated goat anti-rabbit IgG HCS, and pUL10(186–409 aa) was detected by R-HA and HRP-conjugated goat anti-rabbit IgG HCS. (F) Co-IP of pEGFP and pUL10(186–409 aa). (G) Co-IP of pEGFP-UL49.5(1–29 aa) and pUL10(186–409 aa). (H) Co-IP of pEGFP-UL49.5(30–60 aa) and pUL10(186–409 aa). (I) Co-IP of pEGFP-UL49.5(61–95 aa) and pUL10(186–409 aa). HEK-293T cells were cotransfected with relevant plasmids as described in Materials and Methods. M-HA was added to the cell debris to precipitate pUL10(186–409 aa), M-IgG was used as a control, and the antibodies used for WB analysis were the same as for panels D and E. (J) Truncated pUL49.5 promoted the glycosylation of pUL10. DEFs were cotransfected with pCAGGS-UL10-3HA and pEGFP, pEGFP-UL49.5(1–29 aa), pEGFP-UL49.5(30–60 aa), pEGFP-UL49.5(61–95 aa), pEGFP-UL49.5(1–60 aa), and pEGFP-UL49.5(1–95 aa). Cell lysates were collected at 36 h posttransfection and analyzed by WB. M, protein marker.

Based on the results shown in Fig. 2, we searched the domains of interaction between pUL49.5 and pUL10. Subsequently, truncated mutants of pUL49.5 and pUL10 were generated. pCAGGS-UL10(1–185 aa)-HA (the construct contains just aa 1 to 185 of UL10, which is suitable for other similar expressions in this paper) plasmids were transfected into DEFs, but it did not express the target protein (unpublished data); therefore, interactions between pUL10(186–409 aa)-HA and pUL49.5 were examined. The colocalization of pUL10(186–409 aa) and pUL49.5 is shown in Fig. 3A with three different views. HEK-293T cells were cotransfected with pCAGGS-UL10(186–409 aa)-HA and pCAGGS-UL49.5-Flag plasmids, and we found that there was an interaction between them by coimmunoprecipitation (co-IP) and Western blot (WB) analysis (Fig. 3B). To investigate the interaction domains between pUL10(186–409 aa) and pUL49.5 in more detail, pEGFP-UL49.5(1–29 aa), pEGFP-UL49.5(30–60 aa), pEGFP-UL49.5(61–95 aa), pEGFP-UL49.5(1–60 aa), and pEGFP-UL49.5(1–95 aa) were constructed. A model of the truncated UL49.5 is shown in Fig. 3C. pUL49.5(1–29 aa), pUL49.5(30–60 aa), and pUL49.5(61–95 aa) all interact with pUL10(186–409 aa) (Fig. 3D and E), and the results are consistent with those shown in Fig. 3F to I. Taken together, these results indicated that the interaction has multiple sites between DPV pUL49.5 and pUL10. Subsequently, the domains in pUL49.5 that promote pUL10 glycosylation were investigated. pUL49.5(30–60 aa) was the main domain that promoted the mature N-linked glycosylation of pUL10, as shown in Fig. 3J. In summary, DPV pUL49.5 can interact with pUL10 by means of sites and can promote the mature N-linked glycosylation of pUL10, especially the pUL49.5(30–60 aa) domain.

### DPV pUL10 (C54) and pUL49.5 (C46) were crucial residues for complex formation.

**FIG 4:**
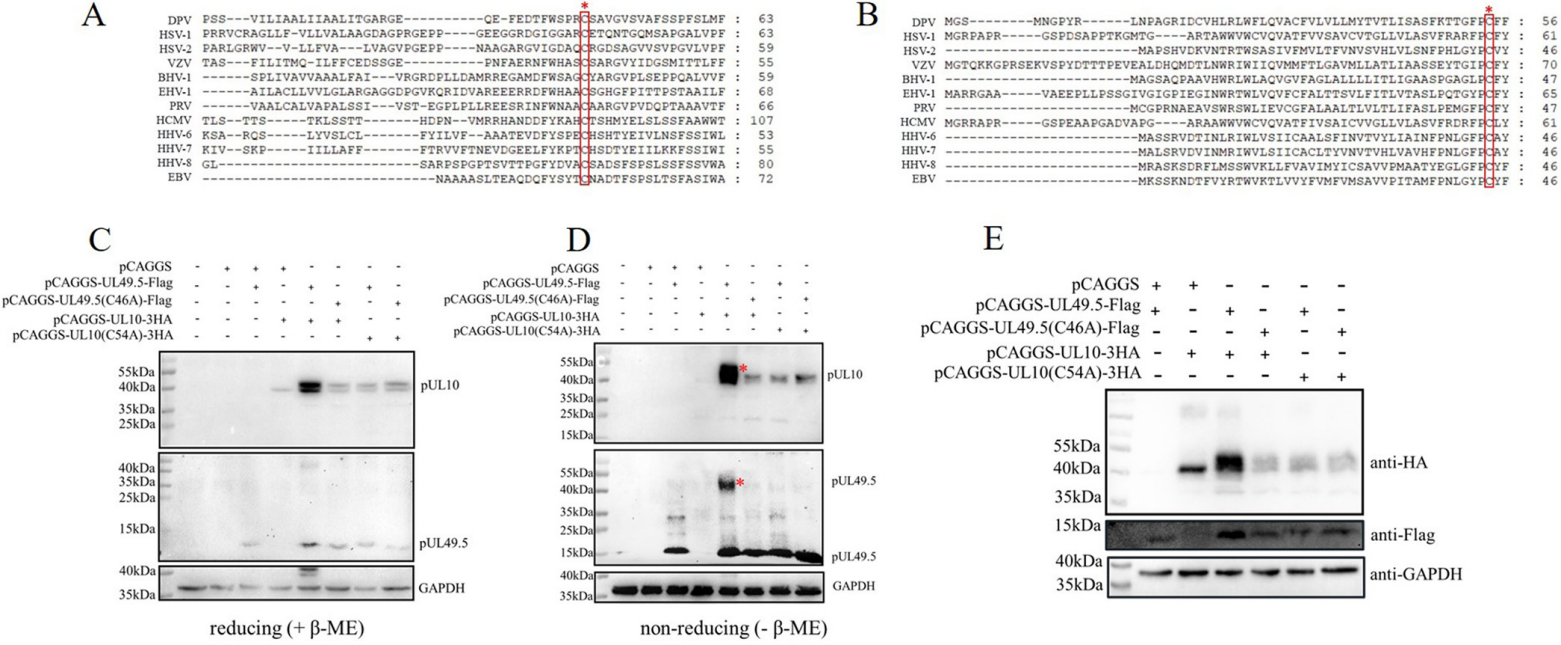
pUL10 (C54) and pUL49.5 (C46) were crucial sites for their interaction. (A) Conservation analysis of DPV pUL49.5 homologs in 12 herpesviruses. (B) Conservation analysis of DPV pUL10 homologs in 12 herpesviruses. Conserved cysteines are marked by red rectangles and asterisks in panels A and B. Information about each sequence used for conservation analysis is shown in Table 1. (C and D) Formation of the pUL49.5/pUL10 complex. Transfection and relevant sample treatments are described in Materials and Methods. Cell debris was collected at 36 h posttransfection and analyzed by WB with reducing and nonreducing SDS-PAGE. The pUL10/pUL49.5 complex is marked by red asterisks. GAPDH was used as a loading control. M, protein marker. (E) pUL49.5 promoted the glycosylation of pUL10. The experimental procedure and antibodies used were consistent with the β-ME treatment experiment.

To explore the interaction between pUL49.5(30–60 aa) and pUL10, we compared their amino acid sites in 12 herpesviruses (Table 1). There were two conserved cysteine residues in pUL49.5 and pUL10, cysteine residue 46 and cysteine residue 54 (C46 and C54), respectively (Fig. 4A and B). The interaction between DPV pUL49.5 and pUL10 may form covalent disulfide bonds because of the two conserved cysteines; therefore, β-mercaptoethanol (β-ME) was used to break intramolecular disulfide bonds. Cell lysates were collected at 36 h posttransfection and treated with or without β-ME (Fig. 4C and D). As shown in Fig. 4D, the molecular weight of pUL10 in the pCAGGS-UL10-3HA and pCAGGS-UL49.5-Flag cotransfection groups was approximately 40 to 45 kDa with β-ME treatment, but it had bands at 40 to 55 kDa (indicated by a red asterisk in the upper panel) without β-ME treatment and not in the other groups. An approximately 50-kDa band of pUL49.5 (indicated by a red asterisk in the lower panel) was detected in the pUL10/pUL49.5 cotransfection group without β-ME treatment, and its molecular mass corresponded with the uppermost image of pUL10 (red asterisk in upper panel). The conserved cysteines were mutated in pUL49.5 and pUL10, and the higher band disappeared in the β-ME-untreated group. The results showed that pUL49.5 C46 and pUL10 C54 formed a covalent disulfide bond. The influence of the two cysteine sites on pUL10 glycosylation was explored. The mature N-linked glycosylation of pUL10 was reduced when pUL49.5 C46 or pUL10 C54 was mutated (Fig. 4C). Additionally, other interaction sites in pUL49.5 still maintained a degree of N-linked glycosylation of pUL10 when the covalent disulfide bond was broken. pUL10 (C54) and pUL49.5 (C46) were vital sites for the formation of the pUL10/pUL49.5 complex.

**TABLE 1:** Information about each sequence used for conservation analysis

Herpesvirus	Abbreviation	DPV UL49.5/UL10 homolog accession no.	Classification
Anatid alphaherpesvirus 1	AnHV-1 (or DPV)	AFC61832.1/AFC61873.1	Alphaherpesviruses
Human herpesvirus 1	HSV-1	O09800/P04288	Alphaherpesviruses
Human herpesvirus 2	HHV-2 (or HSV-2)	Q86539/P89433	Alphaherpesviruses
Varicella-zoster virus	VZV	Q65ZG0/P09298	Alphaherpesviruses
Bovine herpesvirus 1	BHV-1	Q77CE4/P52370	Alphaherpesviruses
Equine herpesvirus 1	EHV-1	P28980/P28948	Alphaherpesviruses
Suid herpesvirus 1	SUHV (or PRV)	Q87088/Q85041	Alphaherpesviruses
Human cytomegalovirus	HCMV	P16795/P16733	Betaherpesviruses
Human herpesvirus 6	HHV-6	Q06094/Q04630	Betaherpesviruses
Human herpesvirus 7	HHV-7	P52366/P52372	Betaherpesviruses
Human herpesvirus 8	HHV-8	YP_001129406.1/YP_001129392.1	Gammaherpesviruses
Epstein-Barr virus	EBV	P03196/P03215	Gammaherpesviruses

### Expression of DPV pUL10 was promoted by pUL49.5.

**FIG 5:**
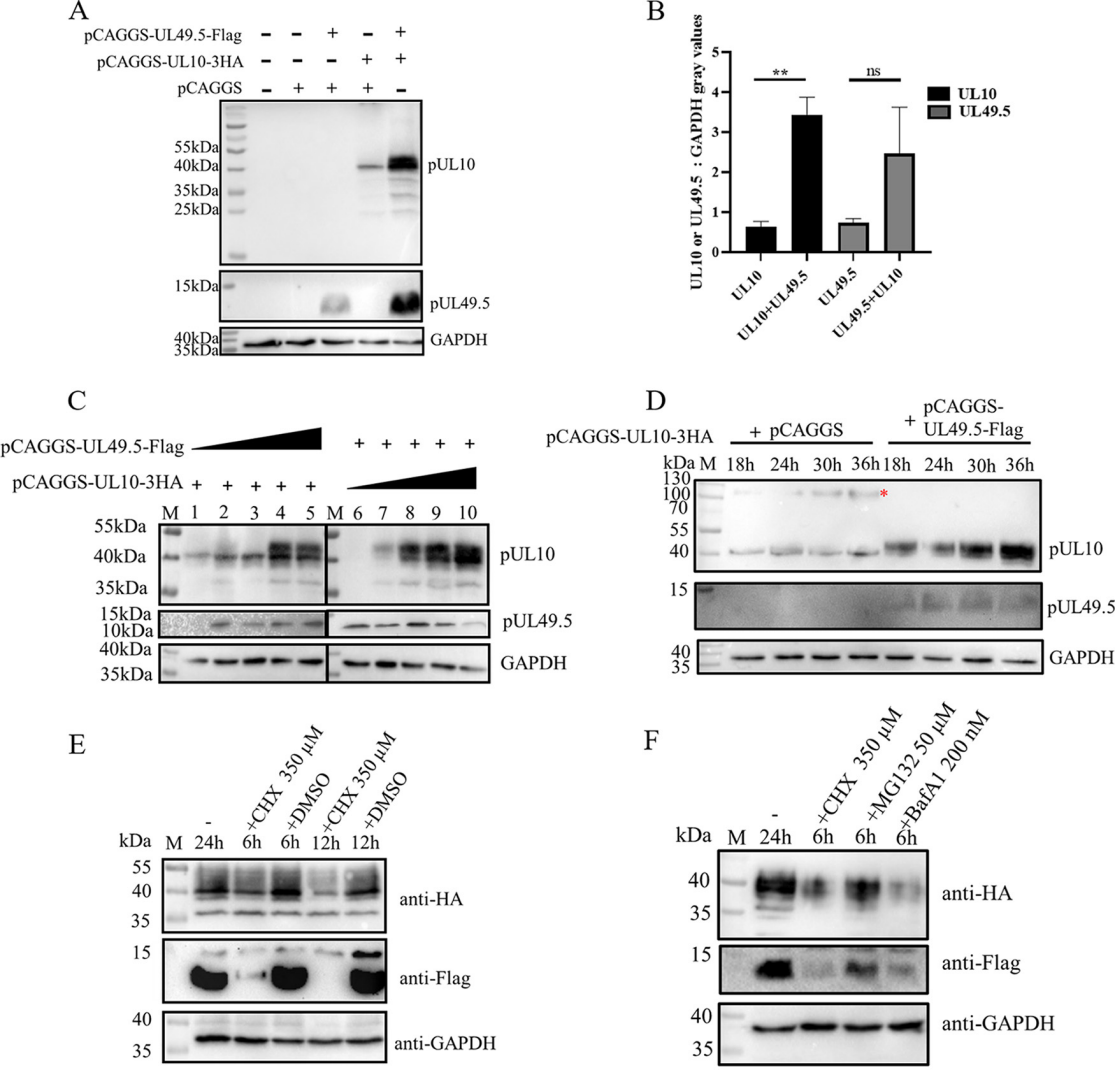
pUL49.5 promoted the expression of pUL10. (A) pUL49.5 promoted the expression of pUL10. DEFs were cotransfected with pCAGGS-UL10-3HA and pCAGGS-UL49.5-Flag. Cell lysates were collected at 36 h posttransfection and analyzed by WB. (B) Gray values of pUL10 or pUL49.5 bands. GAPDH was used as the internal standard. Statistical data are presented as the mean ± SEM. Three independent experiments were performed. Differences between two groups were analyzed using a *t* test; **, *P* < 0.01. (C) DEFs were cotransfected with 1,000 ng of pCAGGS-UL10-3HA and 0, 250, 500, 750, and 1,000 ng of pCAGGS-UL49.5-Flag, and DEFs were cotransfected with 1,000 ng of pCAGGS-UL49.5-Flag and 0, 250, 500, 750, and 1,000 ng of pCAGGS-UL10-3HA. Appropriate amounts of pCAGGS plasmids were supplemented to 2,000 ng per group. Cell lysates were collected at 36 h posttransfection and analyzed by WB. (D) DEFs were cotransfected with 1,000 ng of pCAGGS-UL10-3HA and 1,000 ng of pCAGGS-UL49.5-Flag or 1,000 ng of pCAGGS. Cell lysates were collected at 18, 24, 30, and 36 h posttransfection and analyzed by WB. (E) DEFs were cotransfected with 1,000 ng of pCAGGS-UL10-3HA and 1,000 ng of pCAGGS-UL49.5-Flag. CHX (350 μM) or an equal volume of DMSO was added at 24 h posttransfection. Cell lysates were collected at 24, 30, and 36 h posttransfection and analyzed by WB. (F) DEFs were cotransfected with 1,000 ng of pCAGGS-UL10-3HA and 1,000 ng of pCAGGS-UL49.5-Flag. Then, 350 μM CHX, 350 μM CHX plus 50 μM MG132, and 350 μM CHX plus 200 nM BafA1 were added at 24 h posttransfection, respectively. Cell lysates were collected at 24 and 30 h posttransfection and analyzed by WB. pUL49.5 was measured by M-Flag and HRP-conjugated goat anti-mouse IgG, and pUL10 was measured by R-HA and HRP-conjugated goat anti-rabbit IgG. GAPDH was used as a loading control. M, protein marker.

The expression and molecular mass of pUL10 in the pUL10/pUL49.5 coexpression group were higher than those in the pUL10 and pCAGGS vector cotransfection group, even when the number of pCAGGS-UL10-3HA plasmids in each group was the same (Fig. 4D and E). Therefore, the expression of DPV pUL10 promoted by pUL49.5 was explored. The experimental design is described in Materials and Methods. DPV pUL10 expression was increased when it was coexpressed with pUL49.5 (Fig. 5A). Following densitometric analysis by ImageJ, the ratio of pUL10 and pUL49.5 to GAPDH (glyceraldehyde-3-phosphate dehydrogenase) density was calculated (Fig. 5B). We found that the mature N-linked glycosylation of pUL10 required pUL49.5 participation and that a certain amount of pUL49.5 was needed for more efficient promotion through dose-dependent experiments (Fig. 5C). Additionally, pUL10 expression increased in the presence of pUL49.5 over time, while pUL10 expression was maintained in the pCAGGS group (Fig. 5D). In addition, degradation of pUL10 and pUL49.5 persisted when cycloheximide (CHX) was added to DEFs that were coexpressed with pUL10/pUL49.5 (Fig. 5E). Furthermore, their degradation was prevented by MG132 (proteasomes inhibitor), which means that they were degraded by the ubiquitin-proteasome system (Fig. 5F). Combined with the results above, these results showed that the expression of pUL10 were boosted by pUL49.5.

### Deleting UL49.5 affects the N-linked glycosylation of pUL10.

Based on the above-described experimental results, the effect of pUL49.5 on the N-linked glycosylation of pUL10 in viral infection was explored. Deletion constructs of UL49.5 of BAC-DPV (BAC-DPV-ΔUL49.5) and its revertant virus (BAC-DPV-ΔUL49.5 R) were prepared in our laboratory (34).

**FIG 6:**
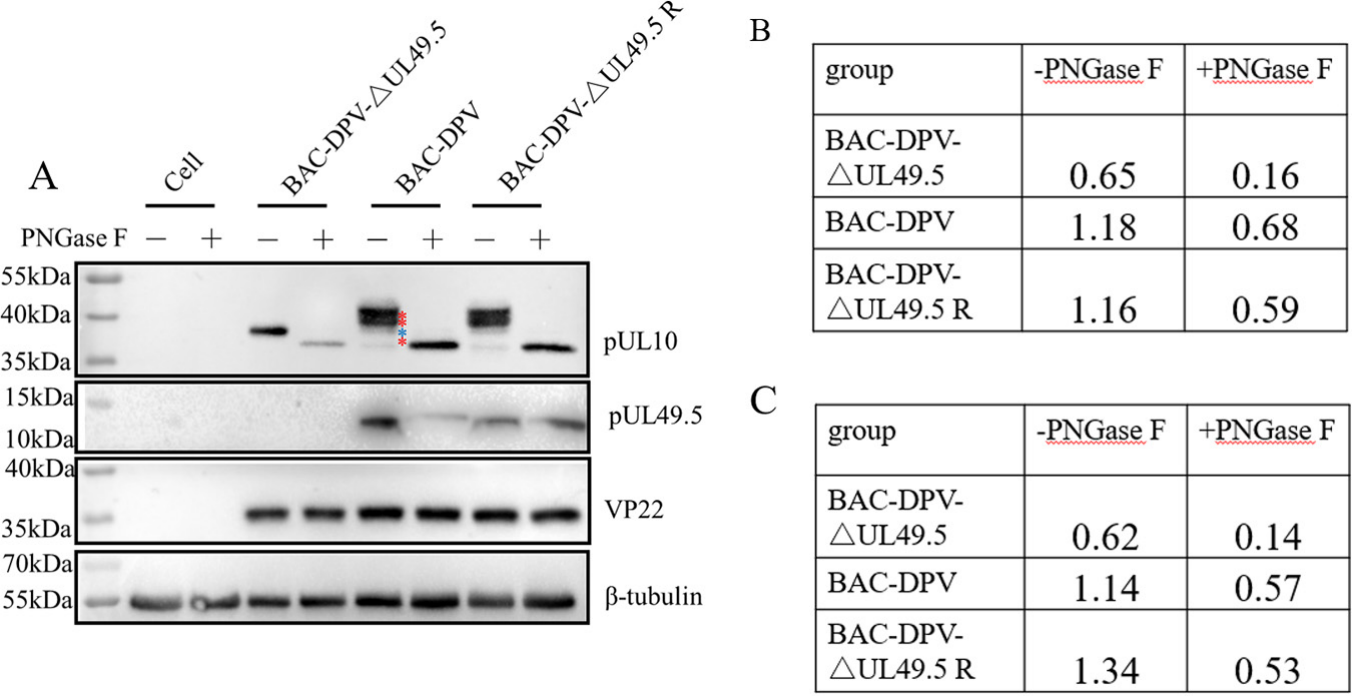
Glycosylation and expression of pUL10 in BAC-DPV-ΔUL49.5-infected cells. (A) DEFs in 6-well plates were infected with 10^6^ TCID_50_ of BAC-DPV-ΔUL49.5, BAC-DPV, and BAC-DPV-ΔUL49.5 R. Cell lysates were collected at 48 h postinfection and clarified by centrifugation. Each group of samples was divided into two parts: one was treated with PNGase F, and the other was not treated. The reactants were analyzed by WB. Rabbit anti-UL10 polyclonal antibodies, rabbit anti-UL49.5 polyclonal antibodies, and rabbit anti-VP22 polyclonal antibodies were used as primary antibodies. HRP-conjugated goat anti-rabbit IgG and HRP-conjugated goat anti-mouse IgG were used as secondary antibodies. VP22 was used as a viral infection control, and β-tubulin was used as a loading control. (B) Following densitometric analysis by ImageJ, the ratio of pUL10 to VP22 density was calculated. (C) Following densitometric analysis by ImageJ, the ratio of pUL10 to β-tubulin density was calculated.

DEFs were infected with equivalent titers of BAC-DPV-ΔUL49.5, BAC-DPV, and BAC-DPV-ΔUL49.5 R. Cell lysates were collected at 48 h postinfection and treated with or without PNGase F to detect the changes in pUL10 molecular mass. pUL10 was concentrated at approximately 36 kDa after treatment with PNGase F in the three strain virus infection groups. Differences were reflected in the untreated PNGase F group. Compared with the molecular weights of the other two virus strain infection groups, the pUL10 molecular weight was decreased by approximately 3 to 10 kDa in BAC-DPV-ΔUL49.5-infected cells, but it was still higher by approximately 2 to 3 kDa than that in the PNGase F-treated group (Fig. 6A). Following densitometric analysis by ImageJ, the ratios of pUL10 to VP22 density and pUL10 to β-tubulin density were calculated, and the results shown in Fig. 6B and C suggest that the expression of pUL10 was reduced in the BAC-DPV-ΔUL49.5-infected cell group compared to the other two groups. According to these results, pUL49.5 affected the expression and mature N-glycosylation of pUL10.

## DISCUSSION

DPV, alternatively named anatid herpesvirus 1 (AnHV-1), is a harmful pathogen for waterfowl. DPV contains a genome, capsid, tegument, and envelope, which together form spherical virus particles. Many protein homologs of the *Herpesviridae* family, such as pUL10 (glycoprotein M homologs), are included in the viral envelope. pUL10 homologs play complex roles in viral fusion, assembly, cell-to-cell spread, immune evasion, and vaccine development (11, 13–21, 27). Since our current knowledge on the biological characterization of DPV pUL10 is limited compared to that of its homologs of other herpesviruses, we focused our study on pUL10 of DPV.

We detected the localization of pUL10 in the cytoplasm, while it was localized in the perinuclear region in DEFs that were infected with BAC-DPV-UL10-HA. In transfection, pUL10 is mainly digested by Endo H and PNGase F, but during infection, pUL10 is mainly digested by PNGase F (Fig. 1), which suggests that other viral proteins promote the mature N-linked glycoprotein, and the site of pUL10 aggregation in the perinuclear region should be the Golgi apparatus, which is the site for maturation of N-linked glycans (35). Interestingly, the bands of pUL10 digested by PNGase F are different in the presence or absence of UL49.5 (Fig. 1B and 2D). pUL10/pUL49.5 complex formation may change the folding of pUL10 and impact the diversity of the glycosylation modification degree in pUL10. Additionally, the pUL10 bands consisted of at least four bands, as indicated by asterisks in Fig. 6A. The blue asterisk indicates the same band as that of BAC-DPV-ΔUL49.5. We speculate that there are other viral proteins that promote band formation, because little difference from the PNGase F treatment group is evident when pUL10 is expressed alone in transfection (Fig. 1B). HA tag causes a little different migration in PNGase F treatment group between Fig. 1G and Fig. 2D and 6A.

DPV pUL49.5 contains three or even more sites to build the connection with pUL10, which is crucial for the conformational stability, transportation, and modification of pUL10. The N-linked glycosylation process is very complicated, and many factors can influence it (36–38). First, the protein requires correct conformation. In some cellular transporters, certain glycosylation sites depend on the formation of intramolecular disulfide bonds, which are also important for maintaining the protein conformation; otherwise, they may lead to functional changes (39). The proteins that can enter the secretory pathway require proper folding in the ER to ensure the native conformation, such as the formation of disulfide bonds (40). As shown in Fig. 5D, pUL10 was labeled with M-HA, and there was a weak band between 100 and 130 kDa (red asterisk), which could be the trimer of pUL10. The trimer would disband due to the intervention of pUL49.5. Therefore, pUL49.5 can affect the conformation and even the activity of pUL10 to boost its expression. N-linked glycosylation on the viral protein surface helps viruses invade host cells and avoid antibody reactions to some viruses (41–45). Correct localization is also critical for protein modification, such as N-linked glycosylation. In HSV-1, pUL10 was present as punctate aggregates around the nucleus in the absence of other viral proteins, and pUL49.5 reached the Golgi apparatus from the ER, which was promoted by pUL10 (17, 46). However, DPV pUL10 is slightly different from HSV-1 pUL10. DPV pUL10 was predominantly localized in the ER when it was expressed alone in DEFs, as was pUL49.5. However, the pUL10/pUL49.5 complex was formed and transported to the perinuclear space. The sensitivity of pUL10 to Endo H and PNGase F changed to a sensitivity mainly to PNGase F (Fig. 1B and 2D), which demonstrated that the mature N-linked glycosylation of pUL10 was completed. In HSV-1, pUL10(1–342 aa) is an Endo H-sensitive protein, but pUL10(1–361 aa) and pUL10 are Endo H-resistant proteins and the localization of these truncated proteins were significantly different from each other (26). N-linked glycosylation requires the N-X-T/S (asparagine-any residue except proline-threonine/serine) sequence. Mutation at any amino acid (N, T/S) would result in the failure of protein glycosylation modification (45). Conversely, the predicted N-linked glycosylation site may not be glycosylated in the natural state, and nonglycosylated sites will be glycosylated when the protein suffers some changes (39).

DPV pUL10 contained two N-linked glycosylation sites according to bioinformatics analysis, and pUL10 was glycosylated during transfection and infection. Kaposi′s sarcoma-associated herpesvirus encodes a homolog of interleukin-6, and it contains two glycosylation sites, asparagine 78 and asparagine 89. The former is present in high-mannose-type N-linked sugars and is dispensable for cytokine function, while the latter affects protein conformation and is critical for receptor binding (47). The HSV-1 pUL10 immature form has been shown to cause limited syncytium formation (48). In BAC-DPV-infected cells, the bands of pUL10 were composed of four parts (marked with red and blue asterisks in Fig. 6A) in the untreated PNGase F group. It showed that pUL10 has various degrees of glycosylation and is mainly dependent on pUL49.5. Each form of pUL10 may serve a different function for virus survival. Therefore, we need to further explore the function of hyperglycosylated pUL10 during viral infection and the influence of pUL10 localization in the Golgi apparatus on viral envelopment.

## MATERIALS AND METHODS

### Cells and viruses.

DEFs were harvested from 9- to 11-day-old duck embryos by 0.25% trypsin (27250018, Gibco) digestion and cultured in Dulbecco's modified Eagle medium (DMEM; 11965092, Gibco) supplemented with 10% newborn calf serum (16010159, Gibco) at 37°C with 5% CO_2_. HEK-293T cells were cultured with RPMI 1640 medium (11875176, Gibco) supplemented with 10% fetal bovine serum (10100147, Gibco) at 37°C with 5% CO_2_. The complete genomic sequence of the DPV CHv strain can be obtained from NCBI (GenBank accession no. JQ647509.1) (4). The whole genomic sequence and an enhanced green fluorescent protein (EGFP) selective marker gene were constructed on the BAC platform, named BAC-DPV, and were stable in Escherichia coli GS1783 (49). BAC-DPV-ΔUL49.5 and BAC-DPV-ΔUL49.5 revertant (BAC-DPV-ΔUL49.5 R) were prepared in our laboratory (34).

### Construction of BAC-DPV-UL10-HA and the recombinant expression vector.

E. coli GS1783 was used for BAC-DPV-UL10-HA construction based on the principle of two red markerless recombinations (50). In brief, the KanR cassette and HA tag were inserted into the carboxyl terminus of the UL10 gene using designed primers that contained homology arms. The KanR cassette was removed due to cleavage of the I-SceI site, and the second recombination occurred in bacteria through a 40-bp sequence duplication. The BAC-DPV-UL10-HA recombinants were extracted using a Qiagen plasmid midi kit (Qiagen, Germany) according to the manufacturer′s recommendations and transfected into DEFs using Lipofectamine 3000 transfection reagent (Invitrogen). Recombinant viruses were cultured and maintained until a large number of green fluorescence plaques were generated. The obtained viruses were identified by sequencing and WB analysis. The HA tag was successfully inserted into the carboxyl terminus of the UL10 gene by sequencing, and the construct was named BAC-DPV-UL10-HA.

DPV UL49.5 (GenBank accession no. AFC61832.1) and Flag tag were cloned into pCAGGS, and the construct was named pCAGGS-UL49.5-Flag. DPV UL10 (GenBank accession no. AFC61873.1) and the 3×HA tag were cloned into pCAGGS, and the construct was named pCAGGS-UL10-3HA. Moreover, eukaryotic plasmids of pCAGGS-UL10(C54A)-3HA, pCAGGS-UL10(186–409 aa)-HA, pCAGGS-UL49.5(C46A)-Flag, pEGFP-UL49.5(1–29 aa), pEGFP-UL49.5(30–60 aa), pEGFP-UL49.5(61–95 aa), pEGFP-UL49.5(1–60 aa), and pEGFP-UL49.5(1–95 aa) were constructed. The modified regions of the recombinant plasmid were checked by sequencing. pCAGGS, pDsRed2-ER, and pEGFP were prepared in our laboratory (51). All designed primer sequences are shown in Table 2.

**TABLE 2:** Primers of BAC-DPV-UL10-HA and the recombinant expression plasmids

Recombinant plasmid and virus	Primer	Primer sequence (5′→3′)
pCAGGS-UL49.5-Flag	UL49.5-F	CATCATTTTGGCAAAATGGCTTCTATGGAGACAG
pCAGGS-UL49.5-Flag	UL49.5-R	TTGGCAGAGGGAAAAAGATCTTTACTTGTCATCGTCGTCCTTGTAGTCCCAATCTACCCTAAACATGTC
pCAGGS-UL49.5(C46A)-Flag	UL49.5(C46A)-F	TCTGGTCGCCCAGAGCATCTGCCGT
pCAGGS-UL49.5(C46A)-Flag	UL49.5(C46A)-R	GCCAACGGCAGATGCTCTGGGCGAC
pEGFP-UL49.5(1–29 aa)	(1–29 aa)-F	GGAAGATCTGCCACCATGGCTTCTATGGAGACAG
pEGFP-UL49.5(1–29 aa)	(1–29 aa)-R	CCCAAGCTTAGTTATAAGTGCGGCGAT
pEGFP-UL49.5(30–60 aa)	(30–60 aa)-F	GGAAGATCTGCCACCATGGGGGCGCGTGGA
pEGFP-UL49.5(30–60 aa)	(30–60 aa)-R	CCCAAGCTTAGAAAAGGGTGACGAGAAG
pEGFP-UL49.5(61–95 aa)	(61–95 aa)-F	GGAAGATCTGCCACCATGCTGATGTTCTACGTAGCTC
pEGFP-UL49.5(61–95 aa)	(61–95 aa)-R	CCCAAGCTTCCAATCTACCCTAAACATGTC
pEGFP-UL49.5(1–60 aa)	(1–29 aa)-F	GGAAGATCTGCCACCATGGCTTCTATGGAGACAG
pEGFP-UL49.5(1–60 aa)	(30–60 aa)-R	CCCAAGCTTAGAAAAGGGTGACGAGAAG
pEGFP-UL49.5(1–95 aa)	(1–29 aa)-F	GGAAGATCTGCCACCATGGCTTCTATGGAGACAG
pEGFP-UL49.5(1–95 aa)	(61–95 aa)-R	CCCAAGCTTCCAATCTACCCTAAACATGTC
pCAGGS-UL10-3HA	UL10-F	CCGGAATTCGCCACCATGGGATCCATGAATGGGCCG
pCAGGS-UL10-3HA	UL10-R	CCGCTCGAGTCAAGCGTAATCTGGAACATCGTATGGGTAAGCGTAATCTGGAACATCGTATGGGTAAGCGTAATCTGGAACATCGTATGGGTATTCACTATCCGACGAGAAATGAGT
pCAGGS-UL10 (C54A)-3HA	UL10 (C54A)-F	ACCGGATTCCCTGCATTTTTTGCCG
pCAGGS-UL10 (C54A)-3HA	UL10 (C54A)-R	ACGGCGGCAAAAAATGCAGGGAATC
pCAGGS-UL10 (186–409 aa)-HA	(186–409 aa)-F	CCGGAATTCGCCACCATGTGTGGAAGAGGAGTAAATAGTGTGACATA
pCAGGS-UL10 (186–409 aa)-HA	(186–409 aa)-R	CCGCTCGAGTCAAGCGTAATCTGGAACATCGTATGGGTATTCACTATCCGACGAGAAATGAGT
BAC-DPV-UL10-HA	DB-UL10-HA-F	TGATACTGTAGCTGAGACTCATTTCTCGTCGGATAGTGAATACCCATACGACGTCCCAGACTACGCTTAGGGATAACAGGGTAATCGATTT
BAC-DPV-UL10-HA	DB-UL10-HA-R	AACTTTTATTATTCAAACCATTCTATAGTTATTATTCTCAAGCGTAGTCTGGGACGTCGTATGGGTATTCACTATCCGACGCCAGTGTTACAACCAAT

### Glycosidase treatment experiment.

To detect the type of pUL10 glycosylation modification in transfection and infection, DEFs in 6-well plates were transfected with 4,000 ng of pCAGGS-UL10-3HA or 2,000 ng of pCAGGS-UL10-3HA plus 2,000 ng of pCAGGS-UL49.5-Flag using 8 μL of PlusTrans transfection reagent (CT801, nulenbiotech; http://www.nulenbio.com/). In some cases, DEFs in 6-well plates were infected with BAC-DPV-UL10-HA (MOI of 1). DEFs in 6-well plates were infected with 10^6^ TCID_50_ of BAC-DPV-ΔUL49.5, BAC-DPV, and BAC-DPV-ΔUL49.5 R for detecting the changes in pUL10 glycosylation modification in the absence of pUL49.5. Cell debris was collected by NP-40 lysis buffer (N8032, Solarbio) at 48 h postinfection or posttransfection and clarified by centrifugation at 12,000 rpm for 15 min at 4°C. PNGase F (P0704S, NEB), PNGase A (P0707S, NEB), Endo H (P0702S, NEB), and O-glycosidase (P0733S, NEB) were used to explore the type of pUL10 glycosylation. According to the manufacturers′ protocol, enzymes and appropriate buffer were added to cell lysates, which were incubated at 37°C for 1.5 h (Endo H, PNGase A, and PNGase F) or 4 h (O-glycosidase). The samples were then subjected to Western blot analysis.

### β-ME treatment experiment.

DEFs in 12-well plates were transfected with 2,000 ng of pCAGGS, 1,000 ng of pCAGGS-UL10-3HA plus 1,000 ng pCAGGS, 1,000 ng of pCAGGS-UL49.5-Flag plus 1,000 ng of pCAGGS, 1,000 ng of pCAGGS-UL10-3HA plus 1,000 ng of pCAGGS-UL49.5-Flag, 1,000 ng of pCAGGS-UL10(C54A)-3HA plus 1,000 ng of pCAGGS-UL49.5-Flag, 1,000 ng of pCAGGS-UL10-3HA plus 1,000 ng of pCAGGS-UL49.5(C46A)-Flag, and 1,000 ng of pCAGGS-UL10(C54A)-3HA plus 1,000 ng of pCAGGS-UL49.5(C46A)-Flag. Cell lysates were collected by NP-40 lysis buffer (N8032, Solarbio) at 36 h posttransfection and clarified by centrifugation at 12,000 rpm for 15 min at 4°C. Each set of samples was divided into two parts: one was treated with β-ME, and the other was treated without β-ME. The samples were identified by WB analysis.

### Co-IP analysis.

To explore the interaction between pUL10 and pUL49.5 in infection and transfection, DEFs in 6-well plates were infected with BAC-DPV-UL10-HA (MOI of 1). In some cases, HEK-293T cells in 6-well plates were transfected with 2,000 ng of pCAGGS-UL49.5-Flag plus 2,000 ng of pCAGGS-UL10-3HA, 2,000 ng of pCAGGS-UL49.5-Flag plus 2,000 ng of pCAGGS-UL10(186–409 aa)-HA, 2,000 ng of pEGFP plus 2,000 ng of pCAGGS-UL10(186–409 aa)-HA, 2,000 ng of pEGFP-UL49.5(1–29 aa) plus 2,000 ng of pCAGGS-UL10(186–409 aa)-HA, 2,000 ng of pEGFP-UL49.5(1–60 aa) plus 2,000 ng of pCAGGS-UL10(186–409 aa)-HA, 2,000 ng of pEGFP-UL49.5(1–95 aa) plus 2,000 ng of pCAGGS-UL10(186–409 aa)-HA, 2,000 ng of pEGFP-UL49.5(30–60 aa) plus 2,000 ng of pCAGGS-UL10(186–409 aa)-HA, and 2,000 ng of pEGFP-UL49.5(61–95 aa) plus 2,000 ng of pCAGGS-UL10(186–409 aa)-HA using 8 μL of PlusTrans transfection reagent (CT801, nulenbiotech). Cell lysates were collected at 36 h postinfection or posttransfection by NP-40 lysis buffer (N8032, Solarbio), and they were clarified by centrifugation at 12,000 rpm for 15 min at 4°C. Mouse anti-HA tag monoclonal antibody (MAb) (M-HA and M180-3, MBL) or mouse anti-Flag tag MAb (M-Flag and M185-3, MBL) was added to the supernatants to immunoprecipitate the bait protein, and they were incubated overnight in a rolling incubator at 4°C. The mouse IgG (M-IgG; A7028, Beyotime) control was analyzed in parallel. Protein A magnetic beads (1614013, Bio-Rad) were added to immunoprecipitate the antibody-bait protein complex. The samples were identified by WB analysis.

### Growth kinetics of BAC-DPV-UL10-HA.

DEFs in 12-well plates were infected with 100 TCID_50_ of BAC-DPV-UL10-HA and BAC-DPV, and supernatants and cells were harvested from three parallel samples at 12, 24, 36, 48, 60, 72, and 96 h postinfection. Cell samples were resuspended in 1 mL of fresh medium and freeze-thawed three times. Viral titers were determined by TCID_50_ assay in DEFs. Subsequently, cytopathic effects were recorded and analyzed. Three independent experiments were performed. Statistical data are presented as the mean ± standard error of mean (SEM). Differences between two groups were analyzed using a *t* test and are presented as a *P* of >0.05 (nonsignificant [ns]), indicating no significant difference.

### IFA.

DEFs in 12-well plates were infected with BAC-DPV-UL10-HA (MOI of 0.1). Infected cells were collected at 16, 24, and 36 h postinfection. DEFs in 12-well plates were transfected with eukaryotic expression plasmids using 3.2 μL of TransIntro EL transfection reagent (FT201-01, TransGen) as follows: 800 ng pDsRed2-ER plus 800 ng of pCAGGS-UL10-3HA, 1,600 ng of pCAGGS-UL10-3HA, 800 ng of pDsRed2-ER plus 800 ng of pCAGGS-UL49.5-Flag, 800 ng of pCAGGS-UL10-3HA plus 800 ng of pCAGGS-UL49.5-Flag plus 800 ng of pDsRed2-ER, and 800 ng of pCAGGS-UL49.5-Flag plus 800 ng of pCAGGS-UL10(186–409 aa)-HA. The DMEM was removed at 36 h posttransfection. For the detailed experimental steps, please see a previous report (52). The rabbit anti-HA tag polyclonal antibody (1:1,000; 51064-2-AP, proteintech), mouse anti-Flag tag MAb (1:500; M185-3, MBL), mouse anti-GM130 (1:100; 610823, BD Biosciences), Alexa Fluor 488-conjugated goat anti-mouse IgG (1:1,000; A-11001, Thermo Fisher), Alexa Fluor 488-conjugated goat anti-rabbit IgG (1:1,000; A-11008, Thermo Fisher), Alexa Fluor 350-conjugated goat anti-rabbit IgG (1:1,000; A-11069, Thermo Fisher), Alexa Fluor 568-conjugated goat anti-rabbit IgG (1:1,000; A-11011, Thermo Fisher), and Alexa Fluor 568-conjugated goat anti-mouse IgG (1:1,000; A-11004, Thermo Fisher) were used. 4′,6-Diamidino-2-phenylindole (DAPI) (62248, Thermo Fisher) was used at 1:1,000 to stain nuclei. The cells were examined under a fluorescence microscope (Nikon, Japan).

### Drug treatment.

DEFs in 12-well plates were cotransfected with 1,000 ng of pCAGGS-UL10-3HA plus 1,000 ng of pCAGGS-UL49.5-Flag using 4 μL PlusTrans transfection reagent (CT801, nulenbiotech). Then, 350 μM CHX (66-81-9, MCE) and isopycnic dimethyl sulfoxide (DMSO) (67-68-5, MCE) were added to the cells 24 h posttransfection. Cell lysates were collected at 6 and 12 h after drug treatment to assess the degradation of the target protein. Cells were treated with 350 μM CHX plus 50 μM MG132 (HY-13259, MCE) and 350 μM CHX plus 200 nM bafilomycin A1 (BafA1) (HY-100558, MCE) for 6 h to identify the protein degradation pathway. Cell debris were subjected to WB analysis.

### WB analysis.

To detect whether pUL49.5 promotes the expression of pUL10, DEFs in 12-well plates were cotransfected with 1,000 ng of pCAGGS-UL10-3HA plus 1,000 ng of pCAGGS-UL49.5-Flag using 4 μL of PlusTrans transfection reagent (CT801, nulenbiotech). To detect the expression of pUL10 by pUL49.5 in a dose-dependent manner, 1,000 ng of pCAGGS-UL10-3HA plasmid was transfected into DEFs, and the amount of pCAGGS-UL49.5-Flag plasmid was 0 to 1,000 ng with an increase every 250 ng in the first group. In another group, 1,000 ng of pCAGGS-UL49.5-Flag was transfected into DEFs, and the amount of pCAGGS-UL10-3HA plasmids was increased from 0 to 1,000 ng every 250 ng. The total amount of plasmids was supplemented to 2,000 ng with the pCAGGS vector plasmid. In the truncated pUL49.5-promoted pUL10 glycosylation assay, 1,000 ng of pEGFP plus 1,000 ng of pCAGGS-UL10-3HA, 1,000 ng of pEGFP-UL49.5(1–29 aa) plus 1,000 ng of pCAGGS-UL10-3HA, 1,000 ng of pEGFP-UL49.5(1–60 aa) plus 1,000 ng of pCAGGS-UL10-3HA, 1,000 ng of pEGFP-UL49.5(1–95 aa) plus 1,000 ng of pCAGGS-UL10-3HA, 1,000 ng of pEGFP-UL49.5(30–60 aa) plus 1,000 ng of pCAGGS-UL10-3HA, and 1,000 ng of pEGFP-UL49.5(61–95 aa) plus 1,000 ng of pCAGGS-UL10-3HA were transfected with the PlusTrans transfection reagent (CT801, nulenbiotech). Cell lysates were collected by radioimmunoprecipitation assay (RIPA) lysis buffer (P0013B, Beyotime) and analyzed by WB. All samples were added to 5× SDS loading sample buffer at a ratio of 4:1. The samples were divided into two parts: one part was incubated at 30°C for 30 min to detect pUL10. The other needed to be boiled for detection of the expression of other proteins. The samples were separated using 15% or 12% separating gels based on the molecular mass of the target protein, and then they were transferred for 40 min or 90 min to a 0.22-μm polyvinylidene fluoride (PVDF) membrane (1620256, Bio-Rad). Then, PVDF membranes were blocked with 5% nonfat powdered milk (A600669, BBI) for 3 to 5 h at room temperature, followed by incubation with primary antibodies overnight and secondary antibodies for 1 h at room temperature. TBST (Tris-buffered saline plus 0.1% Tween 20) was used to wash PVDF membranes before antibody incubation. Commercial antibodies were used as follows: GFP rabbit monoclonal antibody (1:2,000; R-GFP, AF1483, Beyotime), mouse anti-Flag tag MAb (1:3,000; 66008-3-Ig, proteintech), mouse anti-HA tag MAb (1:3,000; M180-3, MBL), mouse anti-GAPDH MAb (1:3,000; 60004-1-Ig, proteintech), mouse anti-β-tubulin MAb (1:3,000; M30109, ABmart), mouse anti-β-actin MAb (1:3,000; 58169, CST), rabbit anti-HA tag polyclonal antibody (1:3,000; 51064-2-AP, proteintech), horseradish peroxidase (HRP)-conjugated goat anti-mouse IgG secondary antibody (1:3,000; SA00001-1, proteintech), HRP-conjugated goat anti-rabbit IgG secondary antibody (1:3,000; 7074S, CST), HRP-conjugated goat anti-mouse IgG light chain secondary (LCS) antibody (1:3,000; A25012, Abbkine), and HRP-conjugated goat anti-rabbit IgG heavy chain secondary (HCS) antibody (1:3,000; A25222, Abbkine). Some antibodies were prepared in our laboratory: rabbit anti-gE polyclonal antibody (1:2,000), rabbit anti-VP22 polyclonal antibody (1:2,000), rabbit anti-pUL10 polyclonal antibody (1:4,000), and rabbit anti-pUL49.5 polyclonal antibody (1:4,000). We used a prestained protein ladder (26616, Thermo Fisher) as an indicator of molecular mass.

### Statistical analysis.

Statistical data are presented as the mean ± SEM. Three independent experiments were performed. Differences between two groups were analyzed using a *t* test, and results are presented as follows: *P* < 0.05 (*), *P* < 0.01 (**), or *P* < 0.001 (***).

### Data availability.

The data sets used during the present study are available from the corresponding author upon reasonable request.
